# Factors Influencing Participation Rate in a Baseline Survey of a Genetic Cohort in Japan

**DOI:** 10.2188/jea.JE20090062

**Published:** 2010-01-05

**Authors:** Megumi Hara, Yasuki Higaki, Takeshi Imaizumi, Naoto Taguchi, Kazuyo Nakamura, Hinako Nanri, Tatsuhiko Sakamoto, Mikako Horita, Koichi Shinchi, Keitaro Tanaka

**Affiliations:** 1 Department of Preventive Medicine, Faculty of Medicine, Saga University, Saga, Japan; 2 Laboratory of Exercise Physiology, Faculty of Sports and Health Science, Fukuoka University, Fukuoka, Japan; 3 Fukuoka Prefectural Government, Asakura Health Welfare Environment Office, Asakura, Fukuoka, Japan; 4 Division of International Health and Nursing, Faculty of Medicine, Saga University, Saga, Japan

**Keywords:** population-based study, survey methodology, response rate, participation rate, reminder

## Abstract

**Background:**

Although many studies have examined factors that influence the response to postal questionnaires, few have addressed baseline recruitment for cohort studies involving genetic analyses. The aim of this study was to describe the method used for a baseline survey, the Japan Multi-institutional Collaborative Cohort Study (J-MICC Study), in Saga Prefecture, and to examine the factors that might influence the recruitment of participants in such studies.

**Methods:**

The Saga J-MICC Study is an ongoing population-based prospective cohort study of the genetic and environmental interactions associated with lifestyle-related disease. From 2005 through 2007, a total of 61 447 residents between the ages of 40 and 69 were invited by mail to participate in this study. The survey date and time were arranged by telephone.

**Results:**

Among that population, 31 002 (50.5%) responded and 12 078 (19.7%) agreed to participate. A completed questionnaire and blood pressure and anthropometric data were collected from all participants; blood, DNA specimens, and accelerometer measures were obtained from the great majority of them. Female sex and older age were associated with a higher participation rate. In addition, the convenience of the survey location and the sending of a reminder significantly improved the participation rate (odds ratio, 1.3).

**Conclusions:**

Our findings suggest that making the survey location as convenient as possible and sending a reminder can both substantially improve participation rate in population-based studies.

## INTRODUCTION

In 2005, we began a population-based prospective cohort study in Saga Prefecture, located in the north of the island of Kyusyu, as part of the Japan Multi-institutional Collaborative Cohort Study (J-MICC Study), which aims to assist in the prevention of cancer and other life-style related diseases by obtaining fundamental data on genetic traits associated with these diseases.^1^ The J-MICC Study is a union of independent cohort studies that are conducted by the Cohort Study Executing Groups (10 groups, including the J-MICC Study, as of April 2008) and is coordinated by the J-MICC central office at the Nagoya University Graduate School of Medicine. The sources of study subject recruitment depend on each study group, and include 1) volunteers residing in areas defined by local governments, 2) participants in health checkups conducted by local governments, 3) visitors to health checkup facilities, and 4) visitors to a cancer hospital.^1^ The target number of study subjects was set at 100 000 throughout Japan, and each study group is expected to enroll more than 5000 participants.

In our study (designated the Saga J-MICC Study), we planned to recruit approximately 10 000 volunteers from residents of Saga City, which had a population of 167 000 as of September 2005. All candidates were enrolled from the resident register and were invited by mail to participate in our baseline survey, which was arranged by us, completely independent of the health checkup program conducted by the local government. Herein, we shall describe the method of the baseline survey and identify the factors that affect participation rate in a community setting. Although many multicenter prospective studies that recruited subjects in such a setting have reported their study design and the characteristics of participants,^2^^–^^7^ few have reported details of the participation rate with respect to methodological factors such as the convenience of the survey location and the sending of a reminder to participants.^8^^–^^10^

## METHODS

### Study subjects and recruitment

On October 1, 2005, the population (and area) of Saga City effectively increased from 167 000 (104 km^2^) to 207 000 (355 km^2^) because of the administrative consolidation of 3 towns and 1 village. Our university, the Saga University Faculty of Medicine, is located in the northwestern part of the former Saga City. We decided to recruit study subjects from residents of the former Saga City because arranging survey locations outside this area was rather difficult, due to the considerable distance between such locations and the university. The former Saga City consisted of 19 that were administratively demarcated to ensure that children could conveniently attend a public elementary school within each area; a public hall is located near each elementary school and these halls were utilized for the baseline survey. Eligible subjects included all residents between the ages of 40 and 69 years living in the above 19 areas; those who could not complete the questionnaire survey or did not give consent to be followed up were excluded. The corresponding target population was approximately 62 000. Assuming a participation rate of 20%, about 12 000 residents were expected to participate.

The study protocol of the overall J-MICC Study was approved by the Ethics Committee at Nagoya University Graduate School of Medicine^1^; the Saga J-MICC Study was approved by the Ethics Committees of both Saga University Faculty of Medicine and Nagoya University Graduate School of Medicine. The baseline survey was conducted from November 1, 2005 through December 22, 2007. For the purpose of convenience, the survey period was divided into 5 phases (November 2005 to March 2006 for the survey of the 3 northern areas, April 2006 to September 2006 for the 5 eastern areas, October 2006 to March 2007 for the 4 southern areas, April 2007 to August 2007 for the 4 central areas, and September 2007 to December 2007 for the 3 western areas). The baseline survey took place during the day, usually on 2 weekdays and 1 weekend, in a public hall within each study area and at other halls available within or outside the area. The survey locations were chosen so that they would be as near as possible to the residential areas of participants, so that they could be easily located by participants. However, such a location was not available in 3 study areas, which were classified as “inconvenient” locations. One such location was outside and distant from the study area, and therefore required longer travel time; the other 2 locations were rather difficult to locate due to their complicated surroundings.

Candidates were enrolled after confirming their name, sex, date of birth, and address on the resident register at the city office. At 1 month before the survey, an invitation letter containing an explanation of the study, a schedule of the baseline surveys at each study area, and a request for participation was sent to the selected subjects. Subjects were asked to reply by mail or facsimile to indicate whether they chose to participate in the study or not. Two weeks after the first contact, a reminder was sent to the subjects who had not responded, in all but 1 study area. Reminders could not be sent to about 40% of the candidates in 1 western area (Nabeshima) where the last surveys had been conducted, because 1 person had strongly complained to city officials about their granting us permission to use personal information in the register, which resulted in him receiving the initial invitation letter and a reminder. Accordingly, those city officials requested that we not send any additional reminders. A schedule of the days of the investigation was arranged by telephone with all subjects who chose to participate. A self-administered questionnaire was sent to the participants beforehand and they were instructed to bring their completed questionnaires to the study site. The questionnaire included a survey of sleeping and exercise, alcohol drinking, smoking, psychological stress, medication and supplements, food intake frequency, family disease history, past disease history, and female reproductive history.

### Baseline survey

Participants gathered in a survey location in each area on the day of investigation. Researchers, who were all medical doctors, orally explained the purpose, contents, and conditions of cooperation of the study, using PowerPoint slides and documents concerning the study; about four to five 30-minute explanation sessions were held per day and, at each session, 1 doctor was assigned to explain the study to each group of approximately 10 to 15 subjects. Informed consent forms were signed by all subjects who agreed to participate, and the forms were all individually checked by either the doctor-in-charge or research nurses. Then, after inspection of the completed questionnaires by research nurses, the participants' blood pressure (systolic and diastolic) and anthropometric characteristics (height, weight, body fat%, and waist and hip circumferences) were measured. A 21-mL blood sample was collected from each participant, using two 6-mL plain tubes for serum, a 7-mL tube with EDTA-2Na for plasma and the buffy coat, and another additional 2-mL tube with EDTA-2K for whole blood. We sent 1.1 mL of serum and 2 mL of whole blood to an external testing laboratory (SRL, Tokyo, Japan) to determine levels of total protein, albumin, triglyceride, total cholesterol, HDL cholesterol, aspartate aminotransferase, alanine aminotransferase, γ-glutamyl transpeptidase, uric acid, creatinine, hemoglobin A1c, and the serostatus of hepatitis B surface antigen and antibody to hepatitis C virus. Within 3 hours, the remaining samples were divided into eighteen 0.3-mL tubes (8 for plasma, 2 for the buffy coat, and 8 for serum) and a 1.5-mL tube (for serum) and stored at −80 °C. Half of the blood samples, except the 1.5-mL tube, were sent to the J-MICC central office. Participants who agreed to allow measurement of their daily physical activities wore an accelerometer (Lifecorder; Suzuken Co., Ltd., Nagoya, Japan) for 10 days, after which they returned them by mail.

### Analyses

The response rate was defined as the ratio of the number of respondents (those who indicated whether they would participate in the study or not) to the number of candidates (those who were invited by mail to attend the study). The participation rate was obtained by dividing the number of participants (those who ultimately participated) by the number of candidates. The response and participation rates were compared by sex, 5-year age, convenience of the survey location, and the receipt of a reminder. Odds ratios (ORs) and their 95% confidence intervals (CIs) for the response or participation with respect to these factors were calculated as measures of the strength of association. When the 95% CIs did not include unity, the corresponding ORs were considered statistically significant if *P* was less than 0.05.

## RESULTS

**Figure 1.**
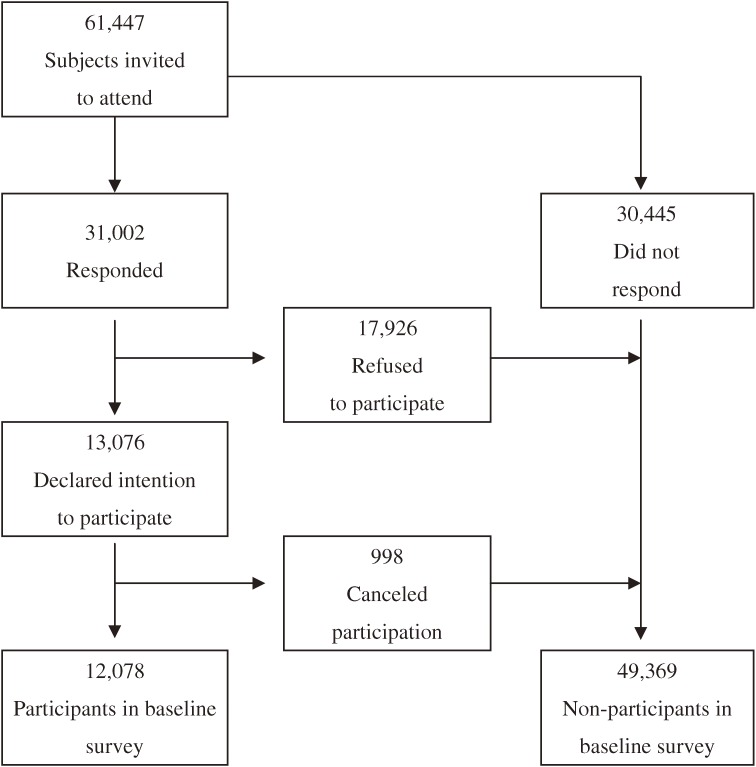
Number of respondents and participants in the Saga J-MICC Study

In total, 61 447 residents of the 19 study areas in Saga City were invited to participate in the Saga J-MICC Study. Of that population, 31 002 indicated whether they would participate or not (response rate, 50.5%). Although 13 076 subjects indicated an intention to participate, 998 of these ultimately canceled; the exact reasons for cancellation were not asked for and thus remain unknown. Consequently, 12 078 subjects participated in this cohort study between November 1, 2005 and December 22, 2007 (participation rate: 19.7%; Figure 1).

Table 1 shows the response rates and participation rates, by sex, age, and the convenience of the survey location. Both the response and participation rates were significantly higher among women than men (OR, 1.3). The rates increased with age: the OR for response in the 65–69 age group, as compared with that in the 40–44 age group, was 2.6 (95% CI 2.5–2.8), and the corresponding OR for participation was 2.0 (1.9–2.2). The convenience of survey location was also associated with both rates, and in subjects with a convenient location, there was a significantly higher response rate (OR, 1.1) and participation rate (1.3) than for those without such a location.

**Table 1.** Response and participation rates in the Saga J-MICC Study, by sex, age group, and the convenience of the survey location

Variable	Invited subjects	Respondents No. (%)	Odds ratio (95% CI)	Participants No. (%)	Odds ratio (95% CI)
Total	61 447	31 002 (50.5)		12 078 (19.7)	
Sex					
Male	29 007	13 549 (46.7)	1 (reference)	5081 (17.5)	1 (reference)
Female	32 440	17 453 (53.8)	1.33 (1.29–1.37)	6997 (21.6)	1.29 (1.24–1.35)
Age at invitation (years)					
40–44	9744	3909 (40.1)	1 (reference)	1491 (15.3)	1 (reference)
45–49	10 108	4334 (42.9)	1.12 (1.06–1.19)	1531 (15.1)	0.99 (0.91–1.07)
50–54	11 082	5212 (47.0)	1.33 (1.25–1.40)	1944 (17.5)	1.18 (1.09–1.27)
55–59	12 750	6596 (51.7)	1.60 (1.52–1.69)	2458 (19.3)	1.32 (1.23–1.42)
60–64	9317	5580 (59.9)	2.23 (2.10–2.36)	2401 (25.8)	1.92 (1.79–2.07)
65–69	8446	5371 (63.6)	2.61 (2.46–2.77)	2253 (26.7)	2.01 (1.87–2.17)
Survey location^a^					
Inconvenient	9500	4610 (48.5)	1 (reference)	1591 (16.7)	1 (reference)
Convenient	49 743	25 703 (51.7)	1.13 (1.09–1.19)	10 129 (20.4)	1.27 (1.20–1.35)

^a^In evaluating the association with the convenience of survey location, 2204 subjects to whom reminders could not be sent were excluded.

CI: confidence interval.

For the purpose of convenience in scheduling the baseline surveys, the Nabeshima area was divided into 2, and letters of invitation were sent first to one section and then to the other. Because the reminders could not be sent to residents in the latter section due to the complaint described in the Methods, the response and participation rates were compared between these sections (Table 2). The response and participation rates in the section to which the reminders were sent were significantly higher than those in the section that did not receive the reminder (ORs for the response and participation: 2.2 and 1.3, respectively).

**Table 2.** Response and participation rates among residents of Nabeshima who did or did not receive a reminder

Variable	Invited subjects	Respondents No. (%)	Odds ratio (95% CI)	Participants No. (%)	Odds ratio (95% CI)
Total	5139	2174 (42.3)		970 (18.9)	
Reminder (−)^a^	2204	689 (31.3)	1 (reference)	365 (16.6)	1 (reference)
Reminder (+)	2935	1458 (50.6)	2.17 (1.93–2.44)	605 (20.6)	1.31 (1.13–1.51)

^a^Reminders could not be sent to these subjects, due to a complaint from a resident, as described in the Methods.

CI: confidence interval.

All participants provided completed questionnaires, as well as blood pressure and anthropometric data. The great majority of them also provided blood (99.9%), DNA specimens (99.7%), and accelerometer data (99.5%); all gave their consent to use these data in the present study (Table 3).

**Table 3.** Characteristics of participants in the baseline survey of the Saga J-MICC Study

	Invited subjects	Participants (%)^a^	Blood provided (%)^b^	DNA provided (%)^b^	Accelerometer data provided (%)^b^
Total	61 447	12 078 (19.7)	12 072 (99.9)	12 041 (99.7)	12 020 (99.5)

Men	29 007	5081 (17.5)	5079 (99.9)	5063 (99.6)	5054 (99.5)
40–44	4625	569 (12.3)	568 (99.8)	563 (98.9)	566 (99.5)
45–49	4776	591 (12.4)	591 (100)	591 (100)	586 (98.2)
50–54	5354	794 (14.8)	793 (99.9)	791 (99.6)	788 (99.2)
55–59	6120	1048 (17.1)	1048 (100)	1044 (99.6)	1044 (99.6)
60–64	4282	1027 (24.0)	1027 (100)	1025 (99.8)	1022 (99.5)
65–69	3850	1052 (27.3)	1052 (100)	1049 (99.7)	1048 (99.6)

Women	32 440	6997 (21.6)	6993 (99.9)	6978 (99.7)	6966 (99.6)
40–44	5119	922 (18.0)	922 (100)	922 (100)	918 (99.6)
45–49	5332	940 (17.6)	940 (100)	939 (99.9)	934 (99.4)
50–54	5728	1150 (20.1)	1150 (100)	1149 (99.9)	1145 (99.6)
55–59	6630	1410 (21.3)	1408 (99.9)	1405 (99.6)	1405 (99.6)
60–64	5035	1374 (27.3)	1374 (100)	1371 (99.8)	1370 (99.7)
65–69	4596	1201 (26.1)	1199 (99.8)	1192 (99.3)	1194 (99.4)

^a^Percentages of proportions to invited subjects.

^b^Percentages of proportions to participants.

## DISCUSSION

The Saga J-MICC Study cohort participants were recruited from community residents in Saga City, a method which has the following advantages. First, the rate of emigration from this rural area is about 4% per year,^11^ which is relatively low in comparison to urban areas of Japan. Therefore, follow-up and retention of study participants is probably easier. Second, this area has a population-based cancer registry,^12^ the Saga Prefectural Cancer Registry, whose data were included in the series, Cancer Incidence in Five Continents Vol. VIII.^13^ This registration will be helpful for the detection of cancer development in future follow-up examinations.

In this baseline survey, the participation rate was 20%, which is substantially lower than the rates for population-based cohort studies in Japan (81%–95%).^3^^,^^5^^,^^14^^–^^16^ In such cohort studies, baseline surveys were most frequently arranged in conjunction with periodic health checkups conducted by local governments. Blood samples were donated in 2 such cohort studies, but donation rates were only approximately 30%.^3^^,^^5^ In the Saga J-MICC study, which was not part of governmental health screening, the participants had to travel to a survey location solely for the purpose of the study, and thus the participation rate may be viewed as relatively high. Several population-based cohort studies, including genetic analyses in foreign countries, reported participation rates of 30% to 78%.^4^^,^^6^^,^^7^^,^^10^^,^^17^^,^^18^ Although it is possible that the participation rate is affected by the study purpose, we do not know whether the genetic aspect of the present study influenced the participation rate. Although many studies have examined the factors that influence the response to postal questionnaires,^9^^,^^19^ few have addressed the baseline recruitment of cohort studies involving genetic analysis.^4^^,^^10^ In this study, female sex and older age were associated with higher response and participation rates. The European Male Aging Study, which collected DNA specimens from men aged 40–79 years, reported that the participation rate was highest among those aged 50–59 years.^10^ In the Prospect-EPIC Utrecht study, the participation rate decreased with increasing age among women aged 50–69 years.^4^ One possible reason for this discrepancy is that, in the present study, older subjects (who were likely to be retirees) and women (who were likely to be housewives) may have been available to attend the baseline surveys during the daytime on scheduled days. Although other demographic, socioeconomic, and lifestyle factors may be correlated with the participation rate,^10^^,^^20^^–^^23^ such information was not available for nonparticipants in the present study.

A main objective of this report was to identify the modifiable factors influencing the participation rate, in order to improve future epidemiological surveys of community inhabitants. In this study, the convenience of survey location and the receipt of a reminder were significantly associated with response rate (ORs: 1.1 and 2.2, respectively) and participation rate (1.3 and 1.3, respectively). Although a literature search yielded no report on the effect of survey location convenience on response and participation rates, several reports have addressed the influence of mailed reminders on the response to questionnaires delivered by post or by hand.^8^^,^^9^^,^^19^ According to a review of 178 articles on mail surveys in 1991,^9^ providing 1 or more reminders with an instrument increased the response rate by 13.8% (OR, 1.8, as estimated by the present authors). In a review of 13 health care studies on patient populations,^9^ a reminder letter had the greatest effect on response rate (3.7). In randomized trials on the effect of reminders, sending reminders to patients increased the response rate by 6% (estimated OR, 1.6) in Denmark and 31% (5.0) in the Netherlands.^8^ In the present study, the improvement in the response rate (OR, 2.2) associated with mailed reminders was comparable to those of the above studies; yet, the ultimate participation rate improved to a lesser degree (1.3). It was uncertain whether this was attributable to the genetic aspect of the present study.

In conclusion, in a population-based cohort study that attempted to identify gene-environment interactions on lifestyle-related diseases, a participation rate of approximately 20% was attained by sending an initial invitation letter with a subsequent reminder to residents of Saga City. Questionnaires, blood pressure, anthropometric information, accelerometer data, blood, and DNA specimens were obtained from the great majority of the participants. This study is expected to produce a great deal of information useful for the prevention of lifestyle-related diseases. Participation rate was associated with the convenience of the survey location and the sending of a reminder. Taking both these factors into account may improve participation rate in population-based studies that include genetic analysis.
